# Study on Laser Drilling of Micro-Holes Using a Breakthrough Detection Method

**DOI:** 10.3390/ma18163764

**Published:** 2025-08-11

**Authors:** Liang Wang, Yefei Rong, Long Xu, Changjian Wu, Kaibo Xia

**Affiliations:** 1 Faculty of Mechanical and Materials Engineering, Huaiyin Institute of Technology, Huaian 223003, China; wangliang@hyit.edu.cn (L.W.); 19816090517@163.com (L.X.); jianjian56791116@163.com (C.W.); 2 School of Mechanical Engineering, Jiangsu University, Zhenjiang 212013, China; xiakaibo@ujs.edu.cn

**Keywords:** laser drilling, millisecond laser, 304 stainless steel, TC4 titanium alloy, penetration detection

## Abstract

Achieving high efficiency and quality in millisecond pulsed laser drilling of metallic through-holes is contingent on precise process control. This study introduces a penetration detection-based method to determine the pulse count threshold, effectively overcoming the limitations of conventional approaches. We systematically investigated the effects of pulse energy, defocus, and beam expansion ratio on the drilling of 3 mm thick 304 stainless steel and TC4 titanium alloy. The experiments revealed that for stainless steel 304, the minimum taper angle was achieved at a pulse energy of 2.2 J, a defocus amount of −0.5 mm, and a beam expansion ratio of 2.5. Additionally, relatively high drilling efficiency was observed when the pulse energy ranged from 2.6 to 2.8 J, the defocus amount was −1 to 0 mm, and the beam expansion ratio was 3 to 4. For titanium alloy TC4, the minimum taper angle was achieved at a pulse energy of 2.6 J, a defocus amount of −0.5 mm, and a beam expansion ratio of 3.5. High drilling efficiency was recorded when the pulse energy was 2.8 J, the defocus amount was −0.5 mm, and the beam expansion ratio ranged from 2.5 to 3. When stainless steel 304 and titanium alloy TC4 were processed using the same laser parameters, the drilling efficiency of stainless steel 304 was higher than that of titanium alloy TC4 under the same conditions. This work provides a practical process control strategy and a valuable parameter database for high-quality, efficient laser drilling of these industrially important metals.

## 1. Introduction

With the rapid advancement of society and industry, micro-holes are being utilized with increasing frequency across a multitude of fields. For instance, these structures play a pivotal role in sectors such as aerospace, medical devices, automotive, and the electronics industry [1,2,3]. A variety of methods are commonly employed for micro-hole fabrication, including micro-drilling [4], electrochemical machining (ECM) [5], electrical discharge machining (EDM) [6], and ultrasonic machining [7]. However, these conventional machining techniques often face limitations that prevent them from fully meeting the demands of modern science and industrial production. In contrast, laser processing has emerged as a highly effective method for creating micro-holes, owing to its distinct advantages such as excellent dimensional control, high precision, non-contact nature, and environmentally friendly characteristics [8,9,10].

A primary challenge in laser drilling, particularly with long-pulse lasers like the millisecond laser, is managing thermal effects that compromise hole quality. These defects commonly include a thick recast layer, significant spatter deposition around the hole entrance, and a large heat-affected zone (HAZ) [11,12]. To address these issues and achieve “cleaner” holes, researchers have explored various advanced assisted technologies. N. Ren et al. (2021) investigated a water-assisted femtosecond laser method for drilling alumina ceramics, demonstrating that water assistance significantly improves hole quality and efficiency by reducing the taper angle and residual debris, particularly at lower pulse repetition rates [13]. H. Zhang et al. (2023) proposed a two-step backside-water-assisted laser drilling strategy using flowing water and employed response surface methodology to optimize the modification parameters, ultimately fabricating a high-quality micro-hole with a taper of only 0.21° [14]. K. Xia et al. (2023) investigated a water-based magnetic assisted method for femtosecond laser drilling of superalloys, demonstrating that this combined approach is superior to individual water or magnetic assistance, significantly improving hole quality by reducing taper and sidewall roughness [15]. Z. Fan et al. (2025) proposed a dual-directional ultrasonic-assisted femtosecond laser drilling technology for film cooling holes, demonstrating with simulation and experiments that this method significantly improves machining efficiency and quality, particularly for inclined holes, by reducing debris deposition and surface roughness [16]. J. Hou et al. (2025) investigated ultrasonic vibration-assisted femtosecond laser drilling of a nickel-based alloy by comparing key processing parameters, demonstrating that ultrasonic assistance improves hole quality with smoother walls and better roundness and identifying an optimal range for ultrasonic power and frequency to achieve this [17].

While these advanced assisted technologies demonstrate significant advantages in improving drilling quality, they also introduce additional complexity, cost, and potential integration challenges into industrial production lines. Therefore, a deep and systematic understanding of the fundamental ‘dry’ laser drilling process remains critically important.

To date, research employing penetration detection technology for a precise analysis of the effects of various laser parameters on the entire through-hole drilling process, especially regarding drilling efficiency, remains limited. Therefore, this study utilizes penetration detection technology to investigate the effects of pulse energy, defocus amount, and beam expansion ratio on the entrance radius, exit radius, and taper angle of through-holes drilled in 304 stainless steel and TC4 titanium alloy. Additionally, the drilling quality and efficiency for both metals are compared, and the differences in the results are analyzed. This work aims to provide a valuable reference for the future laser processing of different metallic materials by optimizing the fundamental process parameters.

## 2. Materials and Methods

### 2.1. Experimental Setup and Materials

The experiments were conducted using a DMG CNC precision laser machining center (DMG MORI, Bielefeld, Germany). This system is equipped with an Nd:YAG laser that generates a Gaussian beam at a wavelength of 1064 nm. The key operational parameters of the laser are as follows: a spot diameter ranging from 0.3 to 1 mm, a pulse duration from 0.1 to 20 ms, and a pulse frequency from 0.1 to 500 Hz. The system’s maximum achievable specifications include an average power of 300 W, a pulse energy of 50 J, and a peak power of 20 kW.

The materials used in this study were 304 stainless steel and TC4 titanium alloy (Baoji Titanium Industry Company Limited, Baoji, China). Both materials were prepared as samples with a diameter of 30 mm and a thickness of 3 mm. Their respective chemical compositions are detailed in Table 1 and Table 2, and their thermophysical properties are presented in Table 3.

### 2.2. Experimental Parameters and Measurement Methods

**Figure 1:**
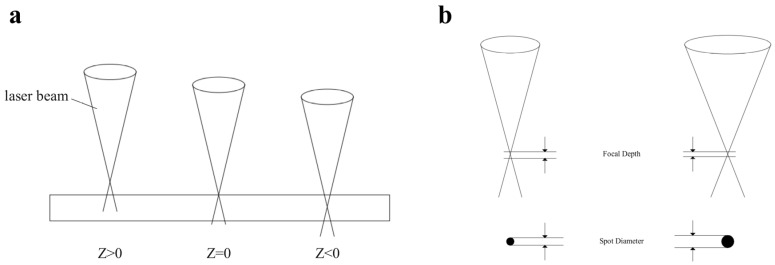
Schematic diagram of key laser processing parameters: (**a**) defocus amount (Z); (**b**) beam expansion ratio.

In this study, the effects of key processing parameters, including the beam expansion ratio and the defocus amount, were investigated. These parameters are schematically defined in Figure 1.

The defocus amount (Z) is defined as the axial distance between the laser’s focal point and the workpiece surface. Three conditions were investigated: positive defocus (Z > 0, focal point above the surface), zero defocus (Z = 0, focal point on the surface), and negative defocus (Z < 0, focal point below the surface). The defocus amounts were set at Z = +1 mm, +0.5 mm, 0 mm, −0.5 mm, and −1 mm.

The beam expansion ratio was controlled using a beam expander. It is defined as the ratio of the output beam diameter to the input beam diameter. The experiments were conducted using beam expansion ratios of 2.0×, 2.5×, 3.0×, 3.5×, and 4.0×.

**Figure 2:**
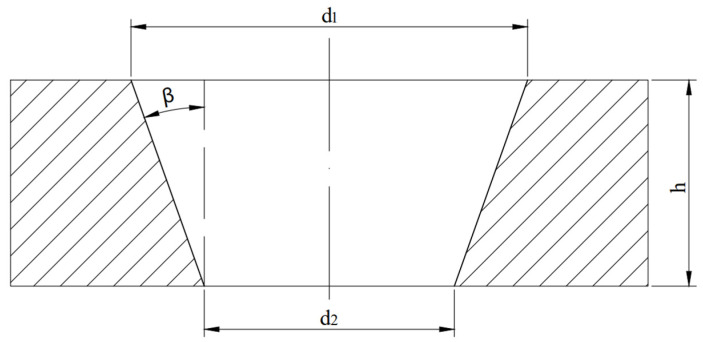
Diagram for calculating the conicity of a through-hole.

For each set of processing parameters, three repeated experiments were conducted to ensure the reliability of the results and evaluate the process stability. The data presented in this manuscript are the average values of these measurements. The taper angle is determined by modeling the through-hole as a truncated cone, as illustrated in Figure 2. In this model, d_1_ and d_2_ represent the entrance and exit radii, respectively; h is the hole depth (equivalent to the material thickness); and β denotes the taper angle. The taper angle is calculated using Equation (1):

(1) $$\beta =\arctan(\frac{d_{1}-d_{2}}{2h})$$

**Figure 3:**
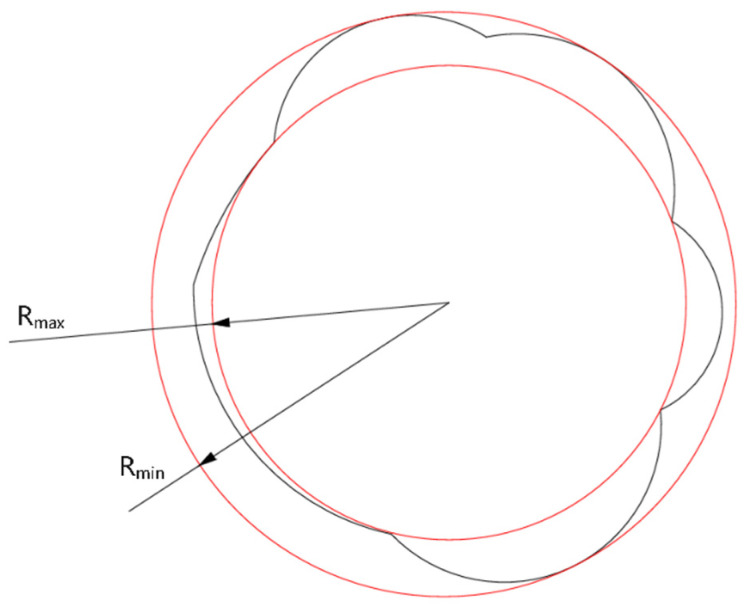
Schematic diagram of roundness calculation.

Figure 3 provides a schematic for the measurement of roundness. In the diagram, the black curve depicts the actual hole profile, while the red concentric circles define its maximum (R_max_) and minimum (R_min_) radii. A smaller roundness value, ΔR, indicates a more uniform and circular hole. The roundness (ΔR) is calculated using Equation (2):

(2) $$\Delta R=R_{\min}-R_{\max}$$

### 2.3. Breakthrough Detection

**Figure 4:**
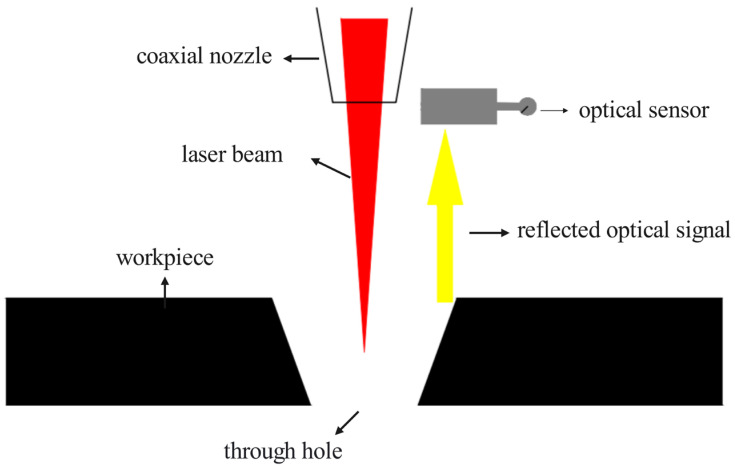
Schematic diagram of the testing device.

Breakthrough detection is the process used to determine if the material has been fully penetrated. As illustrated in Figure 4, this is achieved by monitoring changes in the intensity of the light reflected from the material’s surface. During the laser drilling operation, an optical sensor integrated within the system continuously measures this reflected light intensity. A characteristic change in the reflected signal confirms that the material has been pierced, and a through-hole has been successfully formed.

**Figure 5:**
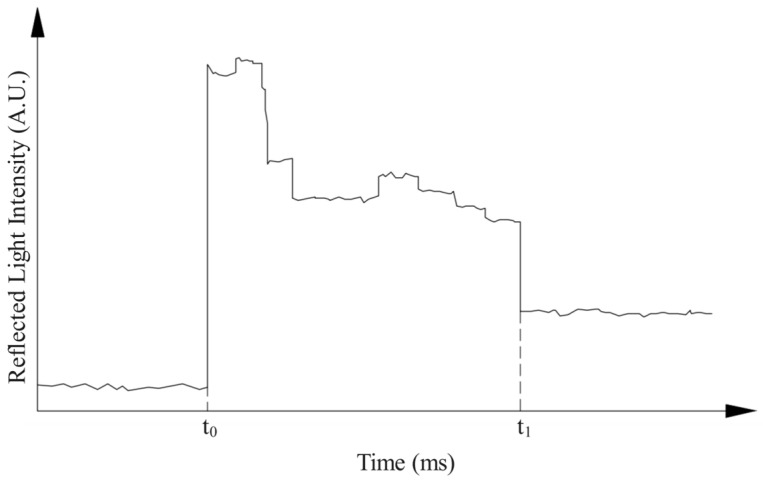
Schematic diagram of a pulse signal.

As depicted in Figure 5, the drilling process commences at time t_0_. The moment of breakthrough, t_1_, is identified by a characteristic change in the reflected light intensity. Given the laser’s pulse repetition frequency, *f*, the threshold number of pulses, N, required to achieve breakthrough can be determined using Equation (3):

(3) $$N=(t_{1}-t_{2})*f$$

## 3. Results

### 3.1. Experimental Results and Analysis for 304 Stainless Steel

#### 3.1.1. Effect of Pulse Energy

Pulse energy is defined as the amount of energy delivered by a single laser pulse. To investigate its impact on the through-hole drilling of 304 stainless steel, the pulse energy was systematically varied while all other laser parameters were held constant. The results of this experiment are presented in Table 4. The materials used in this study were 304 stainless steel and TC4 titanium alloy. Both materials were prepared as samples with a diameter of 30 mm and a thickness of 3 mm. Their respective chemical compositions are detailed in Table 1 and Table 2, and their thermophysical properties are presented in Table 3.

**Figure 6:**
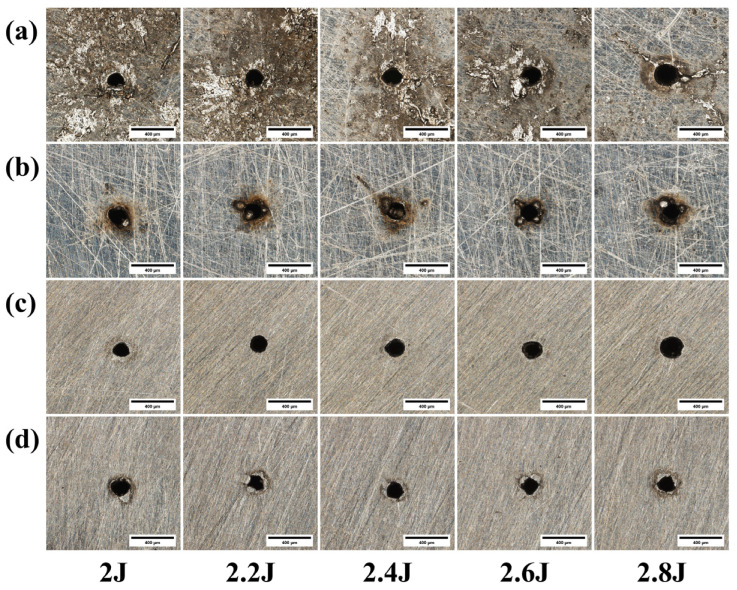
Morphology of 304 stainless steel at different pulse energies: (**a**) entrance before grinding; (**b**) exit before grinding; (**c**) entrance after grinding; (**d**) exit after grinding.

**Figure 7:**
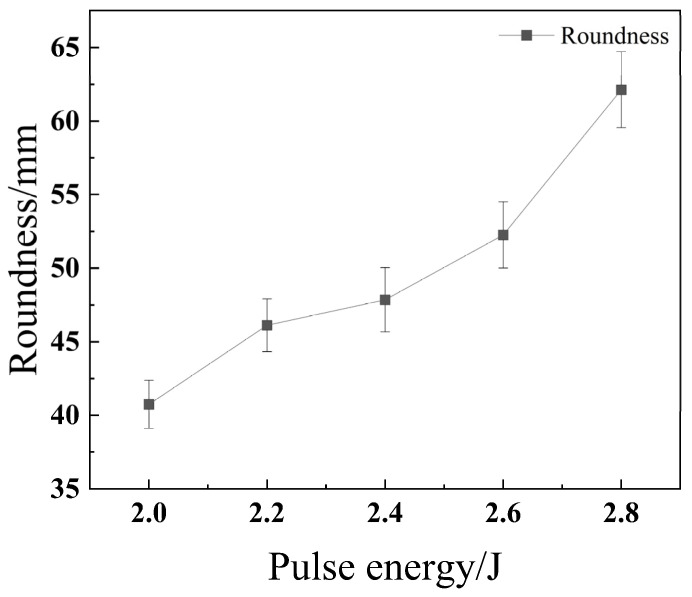
Impact of pulse energy on hole roundness of 304 stainless steel.

**Figure 8:**
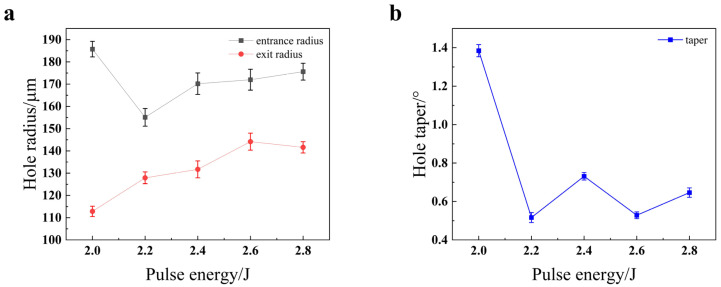
Impact of pulse energy on the quality of the through-hole in 304 stainless steel: (**a**) hole radius; (**b**) hole taper.

Figure 6 presents images of the micro-hole morphologies obtained from laser drilling experiments conducted at various pulse energies. Figure 7 illustrates the effect of pulse energy on the roundness of the micro-holes, while Figure 8 shows its impact on the overall through-hole quality. As observed in Figure 6a,b, the amount of molten material surrounding the hole entrance generally increases with higher pulse energy. Chatterjee et al. (2018) also confirmed in their study on AISI 316 steel that the spatter area increases with laser energy, as the higher heat input melts more material than can be completely flushed away, leading to re-deposition [19]. This is because a greater pulse energy results in more significant melting of the material, which in turn generates a larger volume of molten ejecta. Figure 6c indicates that the hole entrance radius also tends to increase with pulse energy. Furthermore, as shown in the morphology of Figure 6d and plotted in Figure 7, the roundness of the exit hole generally degrades, becoming more irregular as the pulse energy increases.

The influence of pulse energy on the hole geometry, as depicted in Figure 8, is governed by a complex interplay between ablation efficiency and plasma dynamics. Figure 8a reveals a pronounced non-monotonic trend for the entrance radius, which initially decreases to a minimum at 2.2 J before increasing at higher energies. This counterintuitive initial decrease can be attributed to a shift in the dominant material removal mechanism. At lower energy (2.0 J), inefficient melting may dominate, leading to a wide, thermally affected entrance. As the energy increases to an optimal 2.2 J, the vaporization-induced recoil pressure becomes highly effective, driving a clean, axially focused melt ejection that paradoxically reduces the entrance diameter while improving penetration [20]. This transition directly leads to the dramatic reduction of the hole taper to a minimum of approximately 0.5° at 2.2 J, as shown in Figure 8b. Beyond this optimal point, the subsequent increase in both taper and entrance radius is characteristic of the onset of significant plasma shielding. At higher fluences (≥2.4 J), the dense plasma plume formed above the workpiece absorbs a substantial portion of the incident laser energy, leading to overheating at the hole entrance and reduced energy deposition at the bottom [21]. The final “W” shape of the taper curve suggests a delicate balance, where further energy increases may partially overcome the shielding effect, underscoring the need to navigate a narrow processing window to achieve minimal conicity.

**Figure 9:**
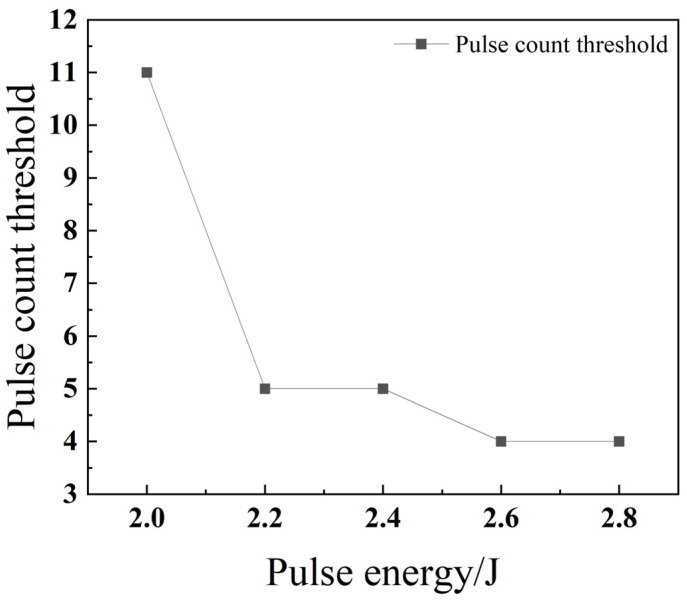
Relationship between pulse energy and the pulse count threshold for 304 stainless steel.

As illustrated in Figure 9, the threshold number of pulses required to achieve through-hole penetration clearly decreases as the pulse energy increases. This is because, when all other parameters are held constant, a higher pulse energy enables each individual laser pulse to melt and eject a greater volume of material. This increased material removal rate per pulse is consistent with findings by Marimuthu et al. (2019), who demonstrated that higher specific energy enhances drilling efficiency [22]. Consequently, fewer pulses are required to drill completely through the material, which in turn leads to a significant improvement in drilling efficiency.

#### 3.1.2. Effect of the Defocus Amount

To investigate its effect, a series of experiments was conducted by varying the defocus amount from −1 mm to +1 mm while all other processing parameters were held constant, as detailed in Table 5.

**Figure 10:**
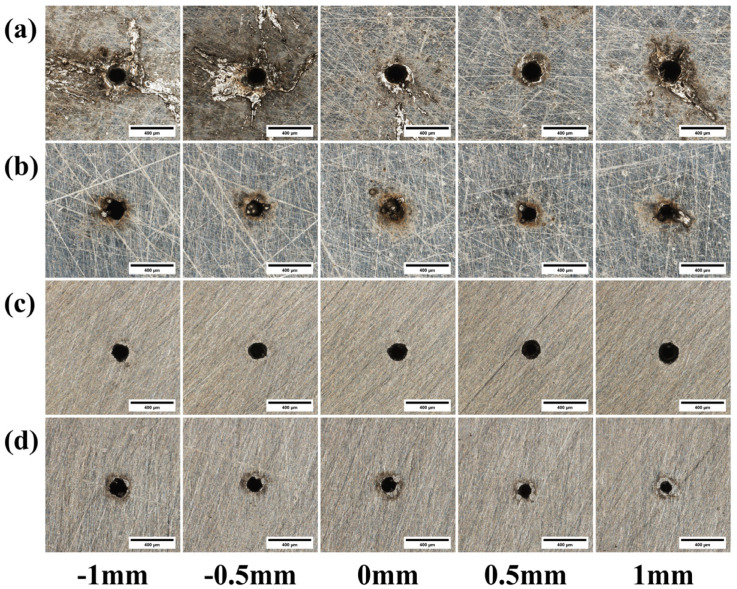
Morphology of 304 stainless steel images with different defocus amounts: (**a**) entrance before grinding; (**b**) exit before grinding; (**c**) entrance after grinding; (**d**) exit after grinding.

**Figure 11:**
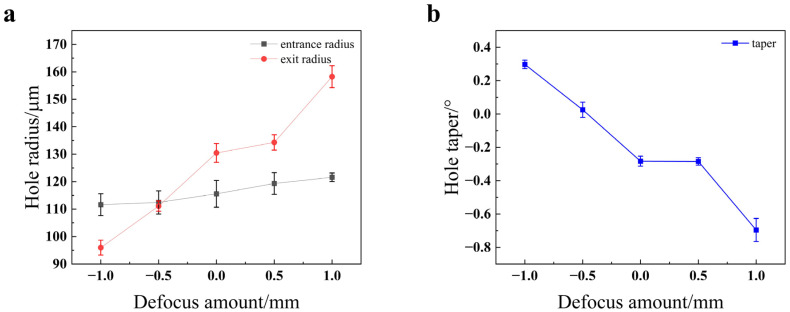
Impact of the defocus amount on the quality of the through-hole in 304 stainless steel: (**a**) hole radius; (**b**) hole taper.

Figure 10 presents the morphology of micro-holes drilled using different defocus amounts, while Figure 11 illustrates the effect of the defocus amount on the quality of the through-holes. Figure 10a reveals that at a positive defocus of Z = +0.5 mm, the amount of molten material accumulated around the entrance orifice is minimal, resulting in the highest hole quality. This is attributed to the fact that the laser’s focal point is positioned slightly above the material surface. This configuration causes the laser beam incident on the surface to be slightly diverged, thereby reducing the spot’s peak energy density and mitigating excessive melting. Conversely, as shown in Figure 10b, under negative defocus conditions, a greater accumulation of molten material is observed at the hole exit. This occurs because the focal point is located near the bottom surface of the material. This positioning concentrates the laser energy at the base, leading to increased melting and a larger recast layer around the exit orifice.

As illustrated in Figure 11, the entrance radius shows little variation with the increasing defocus amount, whereas the exit radius progressively increases. This is because as the defocus amount increases (i.e., moves from negative to positive values), the size of the laser spot on the material’s bottom surface also enlarges, resulting in a wider exit hole. Furthermore, the hole taper is observed to decrease as the defocus amount increases. Notably, the taper reaches its minimum value, approaching 0°, at a defocus of *Z* = −0.5 mm, which indicates the most cylindrical hole profile under these conditions.

**Figure 12:**
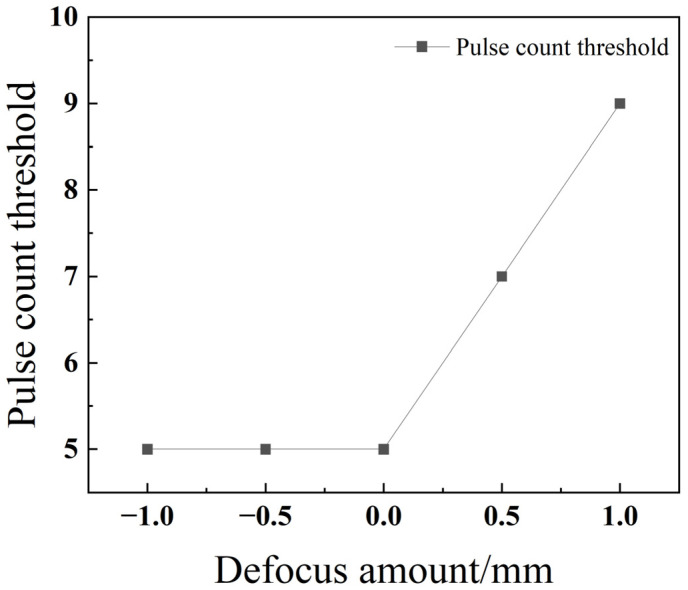
Relationship between the defocus amount and pulse count threshold for 304 stainless steel.

As depicted in Figure 12, the pulse number threshold shows a clear increasing trend as the defocus amount increases (i.e., moves from negative to positive values). This phenomenon can be explained by the differing energy distributions. Under positive defocus, the focal point is above the workpiece, causing the laser beam to be more diverged and dispersed when it strikes the surface. This results in a lower energy density, delivering less effective energy to the material per pulse. Consequently, a greater number of pulses is required to achieve full penetration. In contrast, under negative defocus, the focal point is positioned inside the workpiece. As summarized in the review by Pattanayak and Panda (2018), this configuration concentrates the laser energy within the material, maximizing the energy density at the focal plane [23]. This focused energy is more efficiently utilized for melting and vaporizing material in the longitudinal (depth) direction, promoting an ejection-dominated material removal mechanism that is highly effective for rapid penetration [20]. This facilitates penetration and thereby reduces the required number of pulses.

#### 3.1.3. Effect of the Beam Expansion Ratio

For this study, the beam expansion ratio was varied from 2 to 4 while all other parameters were held constant, as detailed in Table 6.

**Figure 13:**
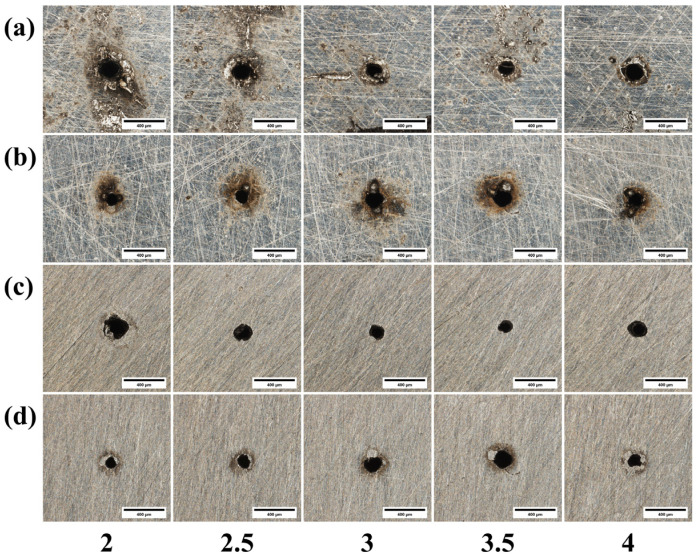
Morphology of 304 stainless steel diagrams of different beam expansion ratios: (**a**) entrance before grinding; (**b**) exit before grinding; (**c**) entrance after grinding; (**d**) exit after grinding.

**Figure 14:**
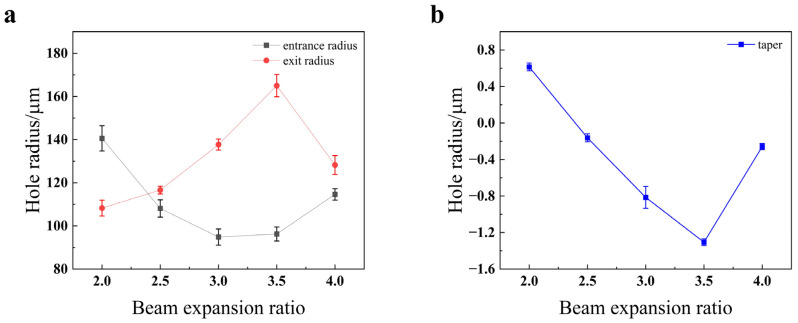
Impact of beam expansion ratio on the quality of the through-hole in 304 stainless steel: (**a**) hole radius; (**b**) hole taper.

Figure 13 presents micrographs of the micro-holes drilled using different beam expansion ratios, and Figure 14 illustrates the corresponding effect on through-hole quality. As shown in Figure 13a, an excessively small expansion ratio leads to a significant accumulation of molten material at the hole entrance, whereas the cleanest entrance with the least dross is achieved at an expansion ratio of 4. This is because a larger expansion ratio minimizes the beam’s divergence before it enters the focusing lens, leading to a higher-quality ablation process at the material’s surface. In stark contrast, Figure 13b,d show that the exit hole quality progressively deteriorates as the expansion ratio increases, with the worst results observed at a ratio of 4. This occurs because a larger expansion ratio, while producing a smaller focal spot, also causes the beam to diverge more rapidly after the focal point, substantially degrading the beam quality and energy density that reach the material’s exit surface. Furthermore, Figure 13c reveals that the entrance radius becomes large at both small and large expansion ratios. At a small expansion ratio, the focal spot itself is larger, naturally increasing the irradiated area and thus the hole diameter. At a large expansion ratio, the small spot’s extremely high energy density enhances lateral energy propagation, leading to greater material removal and a widened entrance.

As illustrated in Figure 14, the entrance radius exhibits a U-shaped trend, initially decreasing and then increasing as the expansion ratio is raised. At first, a larger expansion ratio reduces the focal spot area, which logically results in a smaller entrance radius. However, once the expansion ratio exceeds 3, this trend reverses. This is because when the focal spot becomes sufficiently small, the corresponding energy density becomes excessively high. This extreme fluence can lead to more extensive material melting and lateral heat propagation, widening the entrance hole beyond the geometric spot size [24]. Conversely, the exit radius shows an overall increasing trend with the expansion ratio. This is attributed to the fact that a larger expansion ratio reduces the depth of focus, causing the beam to diverge more rapidly after the focal point and thus creating a larger spot on the material’s bottom surface. A notable exception occurs as the ratio increases from 3.5 to 4; in this range, the extremely shallow depth of focus causes the energy to attenuate rapidly along the depth axis, resulting in the exit hole actually becoming smaller. Finally, the hole taper follows a similar U-shaped pattern, first decreasing to a minimum and then increasing. The optimal (minimum) taper, indicating the most cylindrical hole, is achieved at an expansion ratio of 3.5.

**Figure 15:**
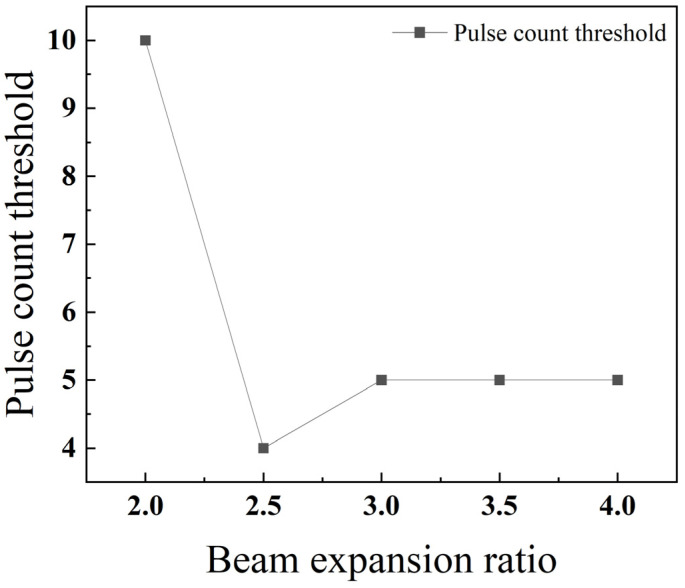
Relationship between the beam expansion ratio and pulse count threshold for 304 stainless steel.

As depicted in Figure 15, the threshold number of pulses required for drilling generally decreases with an increasing beam expansion ratio. A minimum threshold is observed at an expansion ratio of 2.5, indicating the point of maximum drilling efficiency.

### 3.2. Experimental Results and Analysis of Titanium Alloy TC4

Differences in material properties can lead to different laser processing results. To verify the influence of pulse energy, defocus amount, and beam expansion ratio on the entrance radius, exit radius, and taper of through-holes in different materials, the same experiments were conducted on 3 mm thick titanium alloy TC4 based on the experiments with 304 stainless steel, while keeping the experimental parameters unchanged.

#### 3.2.1. Influence of Pulse Energy

**Figure 16:**
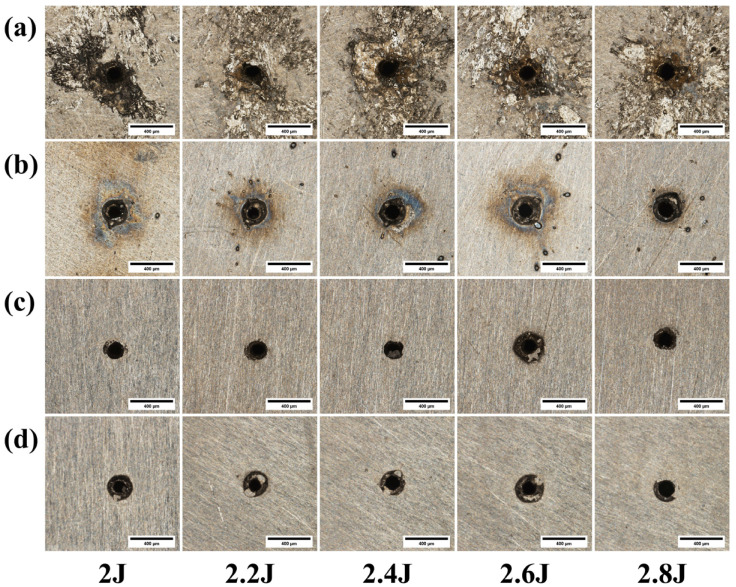
Morphology of TC4 titanium alloy at different pulse energies: (**a**) entrance before grinding; (**b**) exit before grinding; (**c**) entrance after grinding; (**d**) exit after grinding.

**Figure 17:**
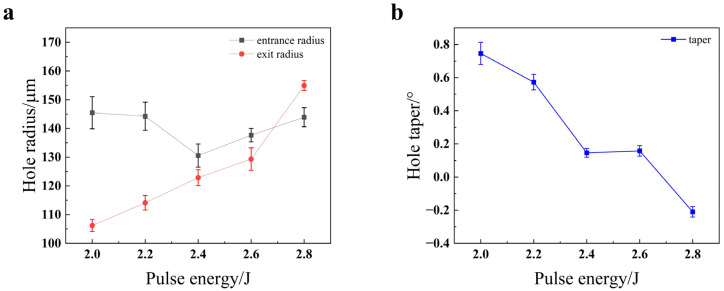
Impact of pulse energy on the quality of the through-hole in TC4 titanium alloy: (**a**) hole radius; (**b**) hole taper.

Figure 16 shows the micrographs of micro-holes from laser drilling experiments under different pulse energies. Figure 17 is a graph showing the influence of pulse energy on through-hole quality. As shown in Figure 16a, compared to 304 stainless steel, titanium alloy TC4 has a greater amount of molten and spattered material on the hole surface due to its higher melting point, making it difficult to observe a clear pattern. Therefore, this aspect of the pre-polishing micro-hole morphology will not be elaborated upon further. As depicted in Figure 16c,d, the entrance and exit radii of the micro-holes increase with the increase in pulse energy.

As shown in Figure 17, the entrance radius decreases at a pulse energy of 2.4 J and then gradually increases beyond this point. This is because higher pulse energy generates plasma, which reduces the material’s absorption of laser energy, thus causing the diameter to decrease at 2.4 J. Subsequently, as the pulse energy continues to increase, the energy’s effective area in the lateral direction expands, leading to a gradual increase in the entrance radius. The exit radius exhibits a trend of increasing in size as the pulse energy rises. This is because as the pulse energy increases, the laser’s penetration depth is enhanced, leading to more uniform heating at the bottom of the hole and thereby enlarging the exit radius. At a pulse energy of 2.6 J, the taper is closest to 0°.

**Figure 18:**
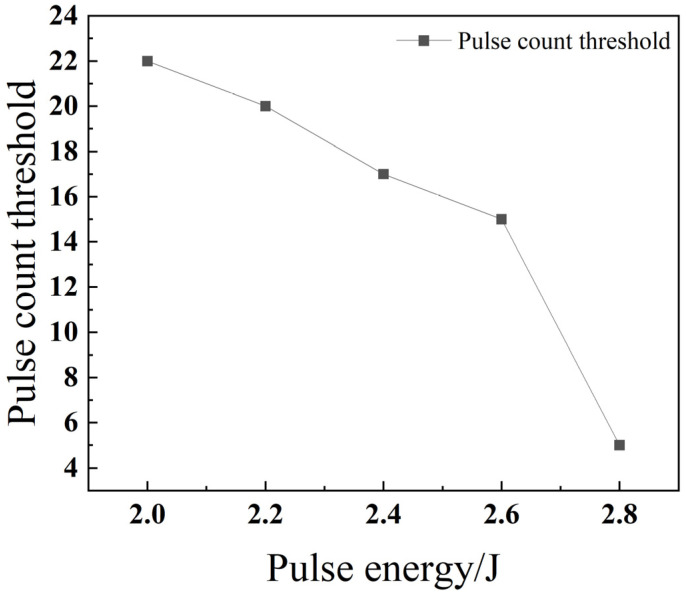
Relationship between pulse energy and the pulse count threshold for TC4 titanium alloy.

Figure 18 shows that the threshold number of pulses decreases as the pulse energy increases, reaching a minimum of five when the pulse energy is 2.8 J. This direct relationship between pulse energy and drilling efficiency is a fundamental aspect of laser percussion drilling, as higher energy density facilitates more rapid material penetration [22].

#### 3.2.2. Effect of Defocus Amount

**Figure 19:**
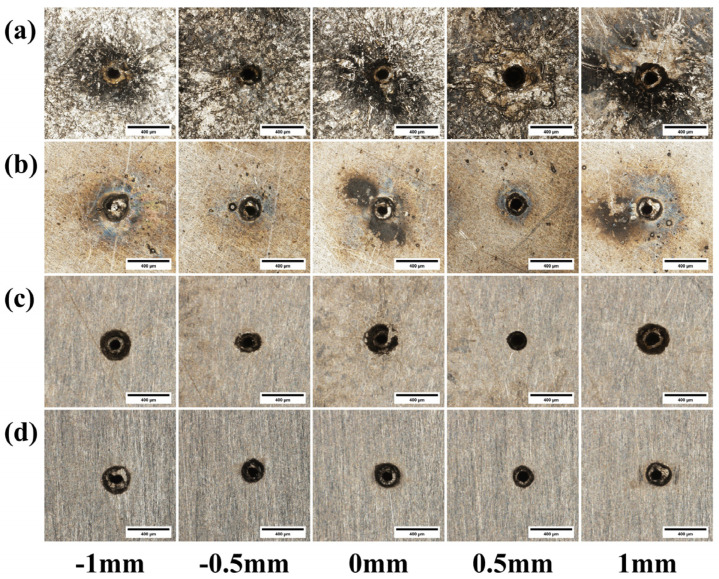
Morphology of TC4 titanium alloy images with different defocus amounts: (**a**) entrance before grinding; (**b**) exit before grinding; (**c**) entrance after grinding; (**d**) exit after grinding.

**Figure 20:**
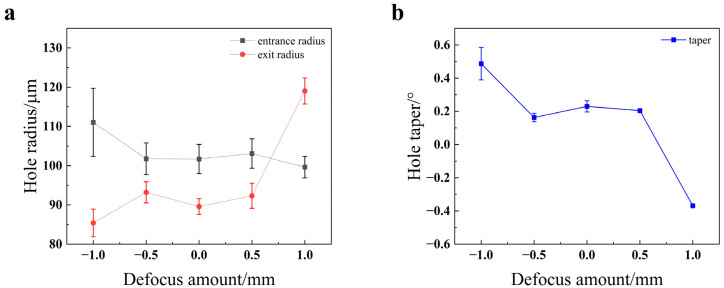
Impact of the defocus amount on the quality of the through-hole in TC4 titanium alloy: (**a**) hole radius; (**b**) hole taper.

Figure 19 shows the micrographs of micro-holes from the laser drilling experiments under different defocus amounts. Figure 20 is a graph showing the influence of defocus amount on through-hole quality. As shown in Figure 19c, when Z = 0.5 mm, the entrance of the micro-hole exhibits a regular shape. At this point, the laser focus is located above the material surface, so the laser beam irradiating the material has a low degree of divergence. This weakens the material removal capability, thereby preventing excessive material removal. As shown in Figure 19d, when Z = 1 mm, the exit orifice is irregular in shape, resulting in the poorest quality.

As shown in Figure 20, the entrance radius decreases as the defocus amount increases. This is because when the defocus amount ranges from −1 mm to 0 mm, the laser’s focal point is located inside the material. The heat generated by the laser at the point of interaction spreads from the surface into the material’s interior, melting the surface layer first and thus leading to a larger entrance radius. When the defocus amount increases from 0 mm to 1 mm, the focal point moves progressively farther from the workpiece surface. This results in reduced energy density on the surface, causing the entrance radius to decrease. The exit radius exhibits an upward trend with the increase in defocus amount. This is because as the defocus amount increases, the area of the light spot at the bottom of the hole becomes larger, thus enlarging the exit radius. As the defocus amount increases, the taper first decreases and then increases, with the taper being closest to 0° at a defocus amount of −0.5 mm. This optimal condition for minimizing taper is a critical finding for achieving high-quality holes and is consistent with studies that seek to balance surface and sub-surface energy deposition [25].

**Figure 21:**
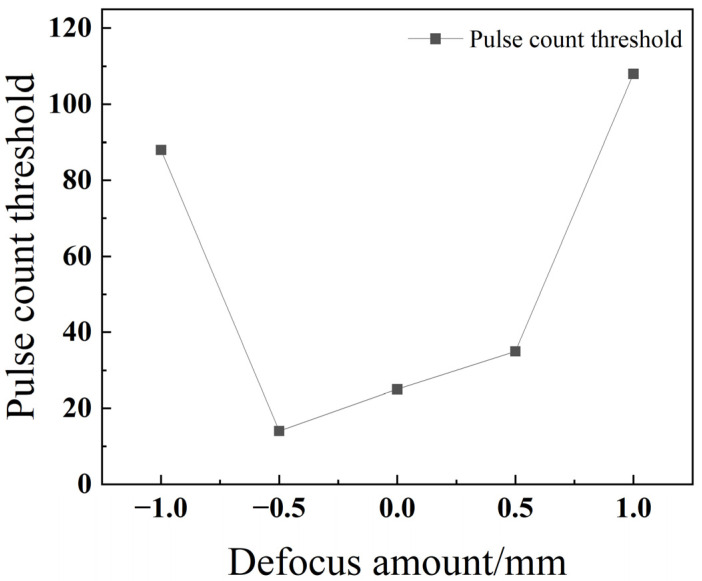
Relationship between the defocus amount and pulse count threshold for TC4 titanium alloy.

Figure 21 reveals that as the defocus amount increases, the threshold number of pulses first decreases and then increases, reaching a minimum at −0.5 mm. This is because with positive defocus, the spot diameter increases, which reduces the laser energy density. In contrast, during negative defocus, when the focal point is located inside the workpiece, the laser’s energy density at the focus is at its highest. This allows more energy to be utilized for melting the material in the longitudinal direction, thereby facilitating penetration during the drilling process.

#### 3.2.3. Influence of Beam Expansion Ratio

**Figure 22:**
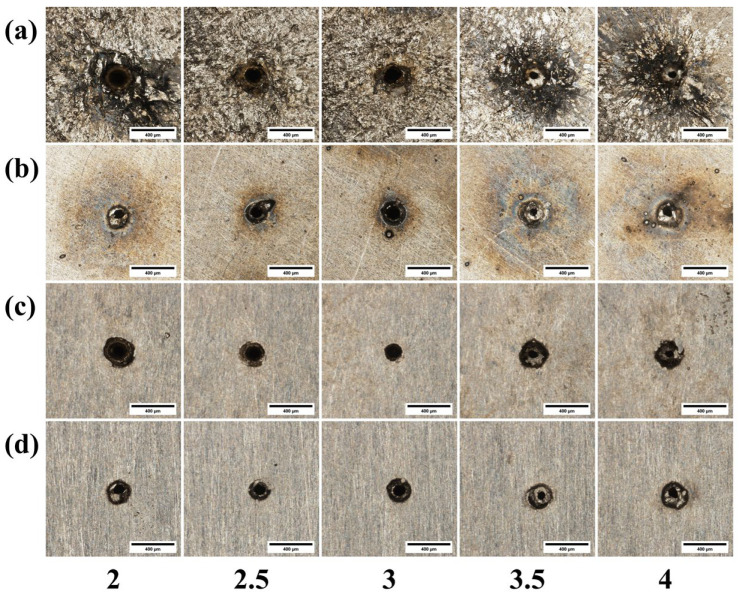
Morphology of TC4 titanium alloy diagrams of different beam expansion ratios: (**a**) entrance before grinding; (**b**) exit before grinding; (**c**) entrance after grinding; (**d**) exit after grinding.

**Figure 23:**
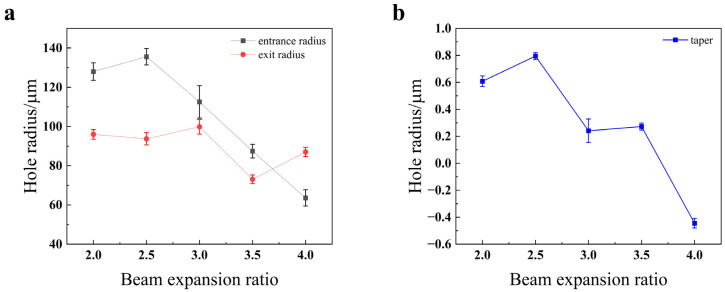
Impact of the beam expansion ratio on the quality of the through-hole in TC4 titanium alloy: (**a**) hole radius; (**b**) hole taper.

Figure 22 shows the micrographs of micro-holes from laser drilling experiments conducted with different beam expansion ratios. Figure 23 illustrates the effect of the beam expansion ratio on through-hole quality. As shown in Figure 22c, when the beam expansion ratio is too small, the entrance radius becomes larger. This is because a small beam expansion ratio results in a large spot area, which in turn increases the irradiated area on the material, leading to a large hole diameter. As indicated by Figure 22d, the overall quality of the exit orifice gradually deteriorates. This is attributed to the fact that as the beam expansion ratio increases, the quality of the light spot at the bottom of the material degrades, resulting in poorer orifice quality.

As shown in Figure 23, the entrance radius exhibits a general downward trend as the beam expansion ratio increases. This is because a higher beam expansion ratio leads to a smaller spot size, which in turn reduces the hole diameter. The exit radius drops at a beam expansion ratio of 3.5. This decrease is attributed to the fact that an increased expansion ratio degrades the beam quality at the bottom of the material, resulting in less effective material removal at the exit orifice. As the beam expansion ratio increases, the taper generally shows a downward trend, with the taper being closest to 0° at a ratio of 3.5. Consistent with this observation, Figure 22c indicates that the hole profile is also optimal at this ratio.

**Figure 24:**
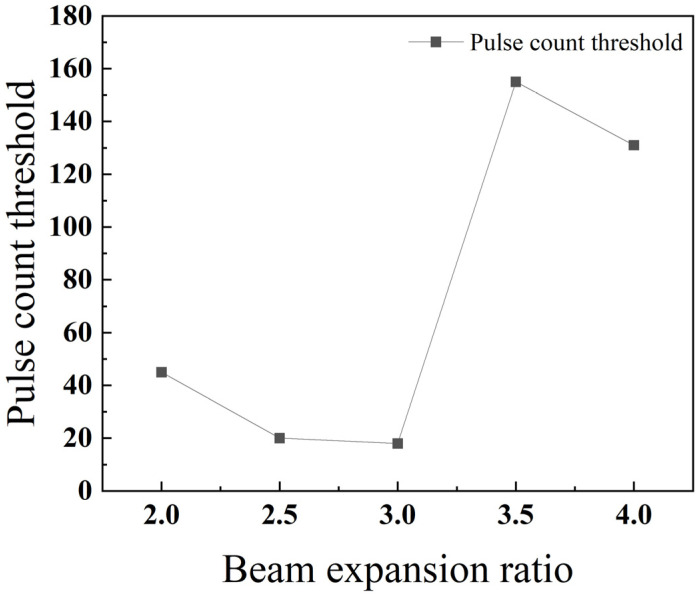
Relationship between the beam expansion ratio and pulse count threshold for TC4 titanium alloy.

As shown in Figure 24, the threshold number of pulses exhibits an overall trend where it first decreases and then increases as the beam expansion ratio grows. This is because a larger beam expansion ratio results in a smaller focal spot area, leading to a higher energy density at the focus and thus greater material removal. However, with an excessively large expansion ratio, the depth of focus becomes very small, and the energy attenuates rapidly in the longitudinal direction, making penetration difficult to achieve. This phenomenon is also observed in the laser machining of TBCs, where residual heating effects reach a quasi-steady state, and further increases in energy density do not proportionally increase the machining rate due to heat conduction and other limiting factors [26].

### 3.3. Comparative Analysis of Stainless Steel 304 and Titanium Alloy TC4

#### 3.3.1. Comparative Analysis of Through-Hole Quality

Taper is a critical parameter in the laser drilling of through-holes, as it directly influences both processing accuracy and quality. In this study, taper is adopted as the primary criterion for evaluating through-hole quality.

**Figure 25:**
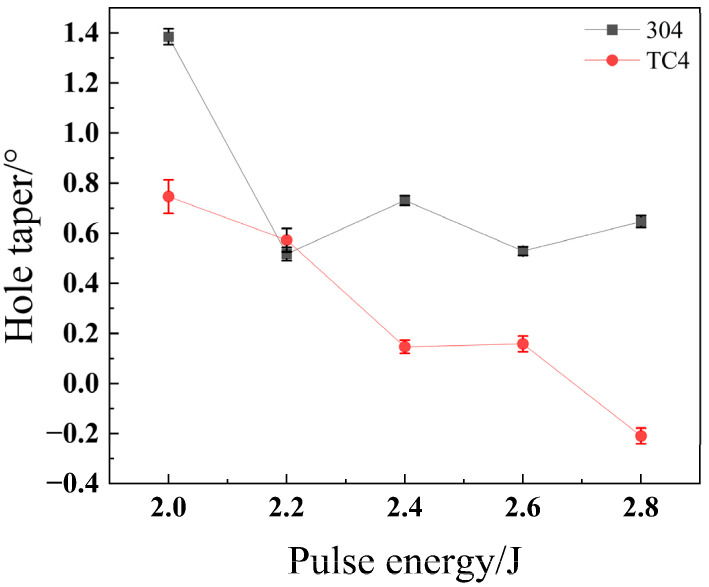
Impact of pulse energy on taper angle for different materials.

As shown in Figure 25, the taper of stainless steel 304 exhibits no clear overall trend as pulse energy increases, showing only minor fluctuations in the 2.2 J to 2.8 J range. Conversely, the taper of titanium alloy TC4 decreases as pulse energy increases. This difference in taper behavior is attributed to the distinct thermophysical properties of the two alloys, a key factor highlighted in reviews of laser drilling [23]. As demonstrated in studies of melt pool dynamics, materials with lower thermal conductivity, like TC4, tend to confine heat more effectively near the interaction zone, while materials with higher thermal conductivity, like 304, allow for more lateral heat dissipation [27]. At low pulse energies, it is difficult for the energy to propagate to the hole’s exit, causing the exit radius to be smaller than the entrance radius. As the pulse energy subsequently increases, more energy reaches the exit, enlarging its diameter and thereby reducing the taper. In contrast, the higher thermal conductivity of 304 may lead to a more balanced energy distribution along the hole depth even at lower energies, resulting in a more stable taper.

**Figure 26:**
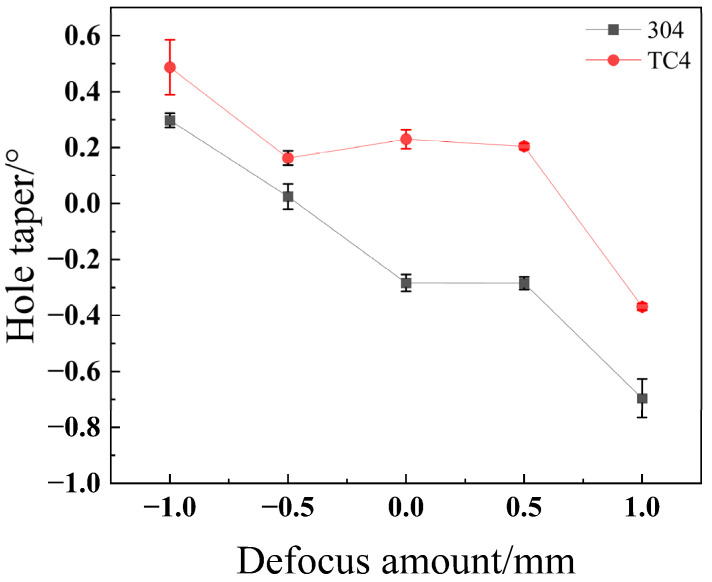
Impact of the defocus amount on taper angle for different materials.

As shown in Figure 26, the through-hole taper for both stainless steel 304 and titanium alloy TC4 steadily decreases as the defocus amount increases (moving from positive to negative values). This phenomenon can be explained as follows: as the focal plane moves from a position above the material to one below it (i.e., as the defocus value shifts from positive to negative), the focal point remains relatively close to the top surface. This proximity ensures that the energy density of the laser spot on the surface remains high, resulting in a relatively minor impact on the entrance hole diameter. Simultaneously, as the focal plane moves deeper, the laser spot incident on the material’s bottom surface expands. This expansion causes the exit radius to increase, and the magnitude of this change is more pronounced than that of the entrance radius. Consequently, this leads to a reduction in the overall taper. Both materials achieve their respective minimum taper at a defocus amount of −0.5 mm.

**Figure 27:**
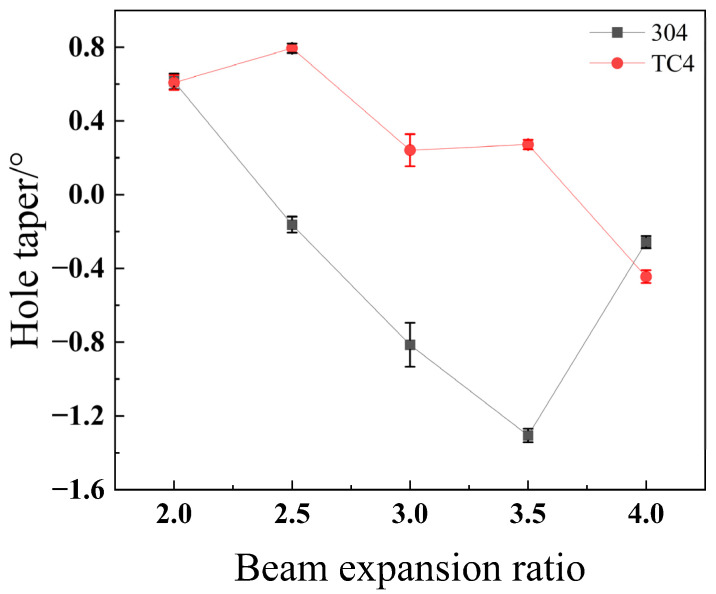
Impact of the beam expansion ratio on taper angle for different materials.

As illustrated in Figure 27, the through-hole taper for both stainless steel 304 and titanium alloy TC4 exhibits a steady downward trend as the beam expansion ratio increases. The underlying reason is twofold. Regarding the entrance hole, an increase in the beam expansion ratio leads to a smaller focal spot diameter, which in turn reduces the entrance radius. Conversely, at the exit hole, as the beam expansion ratio increases, the beam’s divergence angle causes it to directly irradiate the inner wall of the forming hole. This divergent beam becomes the dominant factor in the hole formation process, accelerating the removal of material from the hole wall. This enlarges the exit radius, thereby progressively reducing the hole’s taper. Stainless steel 304 achieves its minimum taper at a beam expansion ratio of 2.5, whereas titanium alloy TC4 reaches an even smaller taper at a ratio of 3.5. The text suggests this difference is because TC4 has a lower thermal conductivity than stainless steel 304, meaning less heat is transferred to the bottom of the material. This is said to result in lower material removal efficiency at the base, which reduces the exit radius and, consequently, the taper.

#### 3.3.2. Comparative Analysis of Through-Hole Drilling Efficiency

The pulse number threshold serves as a key metric for evaluating laser drilling efficiency. A higher pulse number threshold indicates that the material is more difficult to penetrate, corresponding to lower drilling efficiency. Conversely, a smaller threshold signifies that the material is easier to penetrate, resulting in higher drilling efficiency.

**Figure 28:**
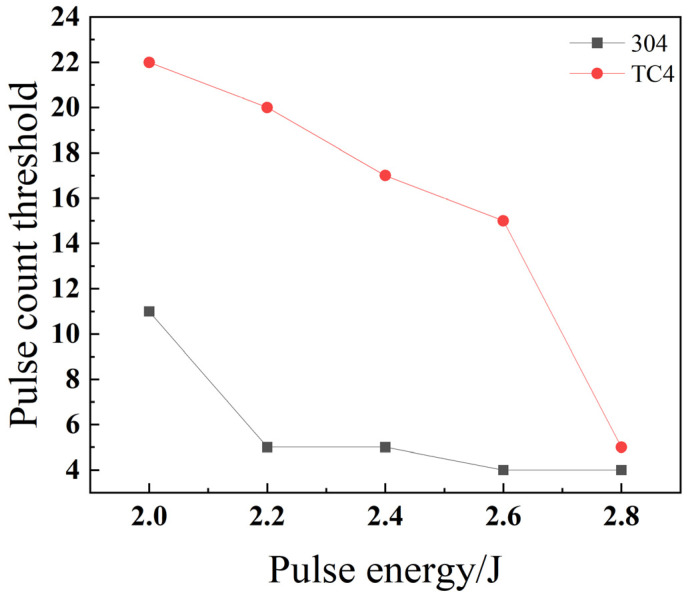
Impact of pulse energy on the threshold of pulse count for different materials.

As shown in Figure 28, the pulse number threshold for both stainless steel 304 and titanium alloy TC4 decreases as the pulse energy increases. This is because an increase in pulse energy leads to higher energy density, allowing each laser pulse to melt a greater volume of material. Consequently, the number of pulses required to penetrate the material decreases, which in turn enhances the drilling efficiency. The pulse number threshold for titanium alloy TC4 is consistently higher than that of stainless steel 304. This difference is attributed to TC4’s material properties: it possesses both a higher melting point and a lower thermal conductivity, which make it inherently more difficult to penetrate compared to stainless steel 304.

**Figure 29:**
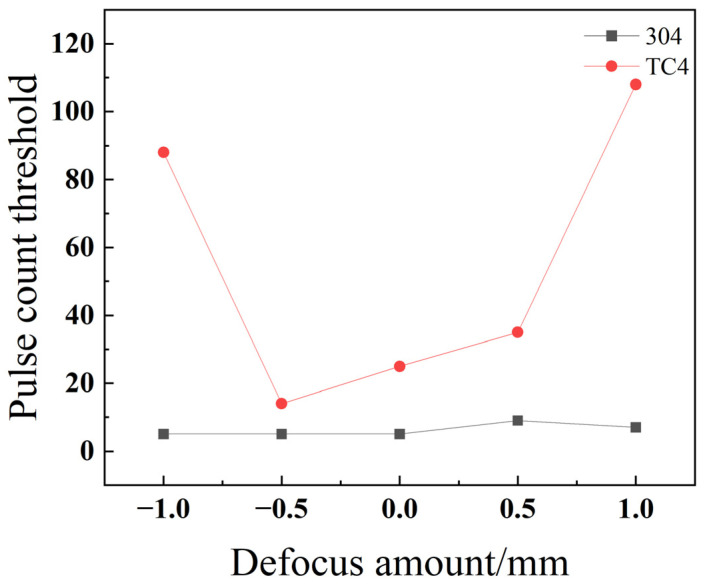
Impact of the defocus amount on the threshold of pulse count for different materials.

As depicted in Figure 29, the pulse number threshold for titanium alloy TC4 follows a parabolic trend as the defocus amount increases: it first decreases, then increases, reaching a minimum value at a defocus of −0.5 mm. In contrast, stainless steel 304 exhibits its lowest pulse number threshold in the negative and zero defocus regions and shows little variation when the defocus amount is altered. This difference in behavior is due to their material properties. Stainless steel 304 has a high thermal conductivity, which allows the laser energy to be rapidly dissipated throughout the material. As a result, its drilling process is less sensitive to changes in the focal plane position. Conversely, TC4’s low thermal conductivity leads to a more pronounced energy accumulation effect on the material’s surface. Consequently, variations in the focal point position have a substantial impact on heat distribution and absorption, causing the pulse number threshold to change significantly. For instance, at a defocus of −1 mm, the focal point is located inside the material. This results in a lower energy density on the material’s surface, meaning more energy accumulation—and thus a greater number of pulses—is required to penetrate the material.

**Figure 30:**
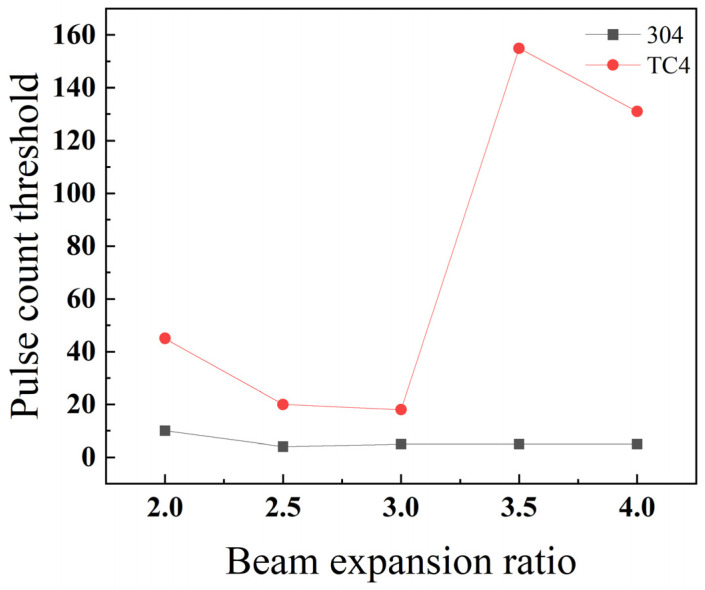
Impact of the beam expansion ratio on the threshold of pulse count for different materials.

As shown in Figure 30, stainless steel 304 achieves its minimum pulse number threshold, and therefore its highest drilling efficiency, at a beam expansion ratio of 2.5. In comparison, titanium alloy TC4 reaches its optimum performance with the lowest pulse number threshold at a beam expansion ratio of 3. However, in the higher range of 3.5 to 4, the pulse number threshold for TC4 becomes significantly greater than that of stainless steel 304. The reason for this divergence is that a large beam expansion ratio results in a very short depth of focus, causing the laser energy to attenuate rapidly along the depth axis. This effect is compounded by TC4’s low thermal conductivity, which hinders the efficient transfer of heat deeper into the substrate. This combination of factors makes the material substantially more difficult to penetrate.

## 4. Conclusions

This study investigated the effects of key laser parameters on the quality and efficiency of millisecond pulsed laser drilling. A primary finding is the inherent trade-off between drilling quality and efficiency; the parameter sets that yield the minimum hole taper (optimal quality) are demonstrably different from those that achieve the fastest penetration (maximum efficiency). Generally, hole quality can be enhanced by optimizing pulse energy toward a moderate level and applying a negative defocus. Beam expansion was found to have a non-monotonic impact on quality, suggesting an optimal ratio exists to balance energy density and spot characteristics. Conversely, drilling efficiency is primarily improved by increasing pulse energy, though often at the expense of hole quality. The successful application of a novel penetration detection technology was instrumental in precisely quantifying these complex relationships, providing a robust methodology for future process optimization in laser drilling.

## Figures and Tables

**Table 1:** Chemical composition of stainless steel 304.

**Composition**	Fe	C	Si	Mn
**Mass fraction/%**	Allowance	0.053	0.46	1.16
**Composition**	P	S	Cr	Ni
**Mass fraction/%**	0.033	0.008	18.15	8.08

**Table 2:** Chemical composition of titanium alloy TC4 [18].

**Composition**	Ti	Al	V	Fe
**Mass fraction/%**	Allowance	6.5~6.8	4.2~4.5	0.3
**Composition**	C	N	H	O
**Mass fraction/%**	0.1	0.05	0.015	0.2

**Table 3:** Thermophysical properties of stainless steel 304 and titanium alloy TC4.

**Thermophysical Properties**	$C/J\cdot g^{-1}\cdot K^{-1}$	$\lambda /W\cdot m^{-1}\cdot K^{-1}$	m.p./°C
**Stainless Steel 304**	0.52	16.3	1400
**Titanium Alloy TC4**	0.612	6.7	1672

**Table 4:** Experimental parameter table for different pulse energies.

Pulse Energy/J	Defocus Amount/mm	Beam Expansion Ratio	Repetition Rate/Hz	Air Pressure/Mpa
2/2.2/2.4/2.6/2.8	0	3	50	0.3

**Table 5:** Experimental parameter table for different defocusing amounts.

Pulse Energy/J	Defocus Amount/mm	Beam Expansion Ratio	Repetition Rate/Hz	Air Pressure/Mpa
2	−1/−0.5/0/0.5/1	3	50	0.3

**Table 6:** Experimental parameter table for different beam expansion ratios.

Pulse Energy/J	Defocus Amount/mm	Beam Expansion Ratio	Repetition Rate/Hz	Air Pressure/Mpa
2	0	2/2.5/3/3.5/4	50	0.3

## Data Availability

The original contributions presented in the study are included in the article, further inquiries can be directed to the corresponding authors.
